# Ocular Surface Ion Transport and Dry Eye Disease

**DOI:** 10.1007/s40135-022-00295-3

**Published:** 2022-10-20

**Authors:** Ethan S. Lindgren, Onur Cil, Alan S. Verkman, Neel D. Pasricha

**Affiliations:** 1 Department of Ophthalmology, University of California San Francisco, San Francisco, CA, USA; 2 Department of Pediatrics, University of California San Francisco, San Francisco, CA, USA; 3 Departments of Medicine and Physiology, University of California San Francisco, San Francisco, CA, USA; 4 Francis I. Proctor Foundation, University of California San Francisco, San Francisco, CA, USA

**Keywords:** Dry eye disease, Ion transport, Ocular surface electrophysiology, Ocular surface epithelium, Cornea, Conjunctiva

## Abstract

**Purpose of Review:**

To review the role of ocular surface epithelial (corneal and conjunctival) ion transporters in the pathogenesis and treatment of dry eye disease (DED).

**Recent Findings:**

Currently, anti-inflammatory agents are the mainstay of DED treatment, though there are several agents in development that target ion transport proteins on the ocular surface, acting by pro-secretory or anti-absorptive mechanisms to increase the tear fluid Film volume. Activation or inhibition of selected ion transporters can alter tear fluid osmolality, driving water transport onto the ocular surface via osmosis. Several ion transporters have been proposed as potential therapeutic targets for DED, including the cystic fibrosis transmembrane conductance regulator (CFTR), calcium-activated chloride channels (CaCCs), and the epithelial sodium channel (ENaC).

**Summary:**

Ocular surface epithelial cell ion transporters are promising targets for pro-secretory and anti-absorptive therapies of DED.

## Introduction

The most recent model of ocular surface ion transporters was published in 2012 [1]. Since then, new therapies and targets have been identified. This review will highlight these developments and put forth an updated model to account for potential novel drug targets.

## Ion Transporters

Ion transporters are proteins that lie in the apical and basolateral membranes of epithelial cells and facilitate the influx and efflux of ions, playing a crucial role in the maintenance of cell homeostasis. The movement of ions across the epithelium establishes transepithelial chemical, electrical, and osmotic gradients that in turn influence water movement [2].

## Mechanisms of Ion Transport

Ion channels use a variety of mechanisms to move ions across the cell membrane. Active transport uses the energy input of ATP hydrolysis to transport molecules between extracellular and intracellular environments against an electrochemical gradient. Passive transport utilizes the potential energy in a concentration or electrochemical gradient. This includes simple diffusion, membrane carrier-mediated facilitated diffusion, and the movement of water through membrane pores, such as aquaporin (AQP). Osmosis is movement of water stimulated by transepithelial osmotic gradients [2].

## Systemic Diseases Due to Ion Transport Dysfunction

Dysfunction of ion transporters can lead to a variety of diseases, and a key example of such is cystic fibrosis (CF). CF is a genetic disease characterized by loss of function mutations in cystic fibrosis transconductance membrane regulator (CFTR), a cAMP-activated chloride channel expressed in many tissues [3]. Dysregulation of CFTR leads to abnormally viscous mucus that results in a variety of comorbidities such as chronic pulmonary inflammation and infection, pancreatic exocrine insufficiency, and male infertility, among others [3]. Of note, some CF patients exhibit low tear film stability on the ocular surface due to the impaired CFTR chloride secretion [4]. As opposed to the loss of function of CFTR in CF, the enterotoxins of *Vibrio cholerae* (producing cholera) and certain *Escherichia coli* strains (traveler’s diarrhea) hyperactivate CFTR causing secretory diarrhea characterized by enhanced salt and water loss in feces [2, 5]. CFTR, along with multiple other ion transporters (Table 1), are expressed on the ocular surface epithelium and play a role in tear production and ocular surface epithelial protection [1, 6, 7••, 8••].

## Dry Eye Disease

The Tear Film and Ocular Surface Society Dry Eye Workshop II (TFOS DEWS II) defines dry eye disease (DED) as, “a multifactorial disease of the ocular surface characterized by a loss of homeostasis of the tear film, and accompanied by ocular symptoms, in which tear film instability and hyperosmolarity, ocular surface inflammation and damage, and neurosensory abnormalities play etiological roles” [9]. DED can be classified as evaporative (e.g. meibomian gland dysfunction), aqueous deficient (e.g. Sjögren’s syndrome (SS)), or mixed. DED is estimated to affect 6.8% of adults in the United States. The global prevalence is estimated to be as high as 50%, with estimations reaching as high as 75% in specific populations [10]. Tear hyperosmolarity and instability are major offenders in DED by damaging the epithelia directly, inducing inflammation, and causing insufficient tear production and excessive tear evaporation [10]. Most FDA-approved therapies for DED target only the inflammation and have limited clinical efficacy [11–13]. The ocular surface, comprised of the cornea and conjunctiva, is lined by stratified epithelial cells expressing ion transport proteins that facilitate fluid secretion or absorption to regulate tear fluid volume and osmolarity. These ocular surface ion transporters thus present an attractive target to develop drugs to treat DED.

## Ocular Surface Ion Transporters

Major ion channels that are functionally expressed in ocular surface epithelial cells include the CFTR chloride channel and the epithelial sodium channel (ENaC) on the apical membrane, which are involved in fluid secretion and absorption, respectively [14–18]. Additional ion channels expressed on the apical membrane include calcium-activated chloride channels (CaCCs), and potassium channels. The basolateral membrane (facing the corneal stroma) expresses potassium channels, an electroneutral Na^+^/K^+^/2Cl^−^ cotransporter (NKCC1), and a sodium-potassium pump (Na^+^/K^+^-ATPase), the latter providing the energy to drive fluid secretion. There is paracellular ion transport as well. To create the electrochemical driving force for apical chloride secretion, and hence fluid secretion into the tear film, the basolateral membrane transporters act in concert to maintain a cell interior negative membrane potential and a high concentration of potassium in the cytoplasm, a low concentration of sodium, and a concentration of chloride that is above its electrochemical equilibrium potential for its transport onto the ocular surface when CFTR or CaCCs are open [7••].

**Fig. 1:**
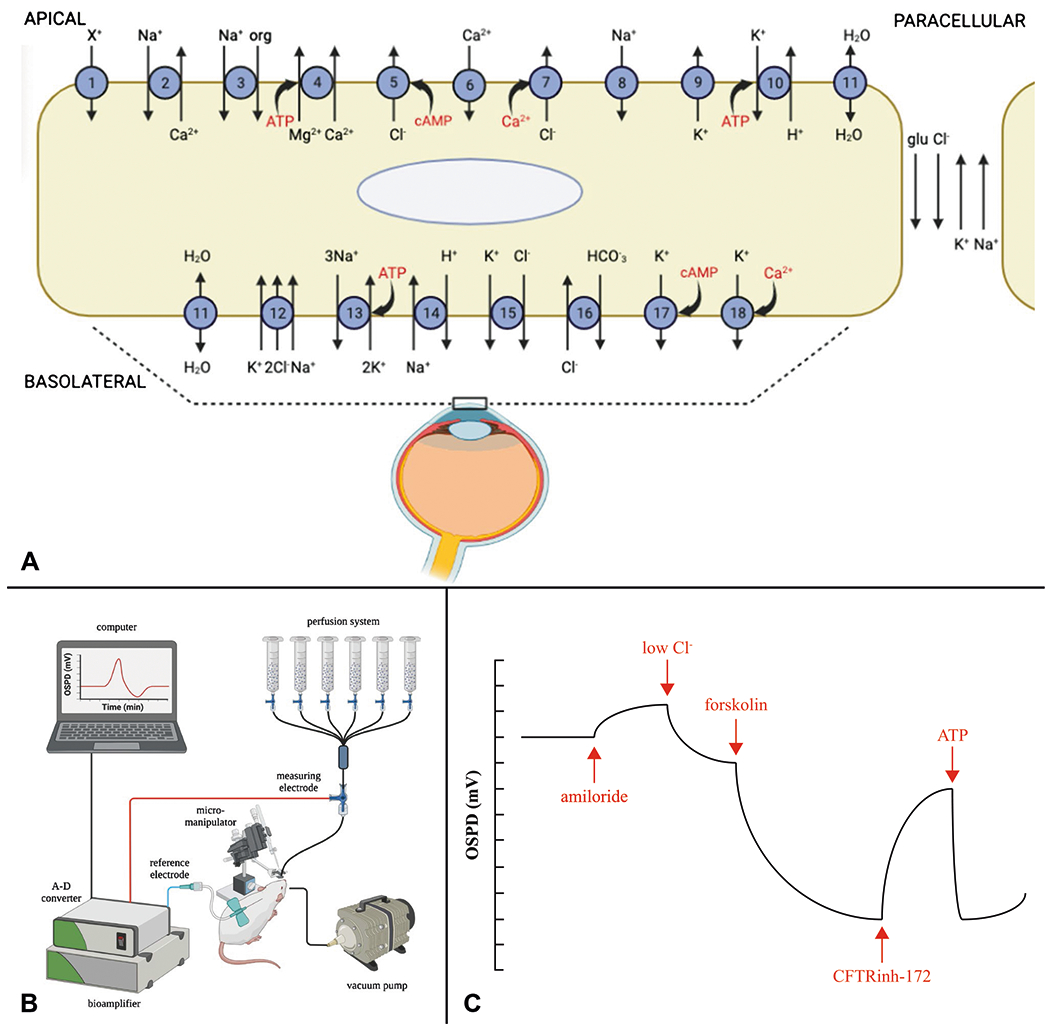
**A** Schematic diagram of membrane proteins in the ocular surface epithelium (cornea and conjunctiva) involved in the movement of ions via ion channels, pumps, transporters, and receptors [1, 16]. See Table 1 for detailed descriptions. **B** Schematic of OSPD setup showing the multi-syringe perfusion system to deliver fluid to bathe the ocular surface and the electrical system with measuring electrode in contact with the ocular surface (through the perfusate), subcutaneous reference electrode, and high impedance bioamplifier to measure the mV electrical potential. **C** Representative OSPD tracing showing PD changes from sequential perfusion exchanges in a typical protocol to study chloride transport

Our updated diagram of ion transporters on the ocular surface epithelium includes the original ion transporters recognized in 2012 with the addition of P2Y2, ASIC, Clc-2, and additional potassium channels (Table 1 and Fig. 1A) [1]. Transporter gene names, locations (apical or basolateral), and putative functions are listed in Table 1. The roles of CFTR and ENaC in tear fluid secretion and absorption, respectively, are well established. However, less is known for the other ion transporters. NHE8, a Na^+^/H^+^ exchanger expressed on the basolateral membrane, is suggested to play a role in tear production and ocular epithelial protection [19]. Clc-2, a non-CFTR cAMP-activated chloride channel expressed on the apical membrane, may play a similar chloride secretory role to CFTR in many tissues, including the eye [20]. ASIC, a voltage-insensitive acid-sensing cation (mainly sodium) channel, is implicated in increasing tear and blinking rates and reducing ocular neuropathic pain [8••]. While their exact etiologic roles remain unclear, various potassium channels (e.g. voltage-gated, cAMP-activated, and calcium-activated) are expressed in the ocular surface epithelium and may also play a role in lacrimal gland secretion [21].

## Therapeutic Ocular Surface Ion Transport Targets

Currently, there are no FDA-approved drugs for DED that target ion transporters. However, there are several drugs in various stages of clinical development including drugs approved outside the USA. Pro-secretory drugs include VSJ-110, a small-molecule triazine CFTR activator with nanomolar potency currently in phase 2 clinical trial. VSJ-110 stimulates secretion of chloride from the apical membrane and has been shown to increase tear secretion and reverse epithelial damage in mice and rabbits [22•, 22–26]. Another pro-secretory drug is diquafosol, a purinergic P2Y2 agonist currently approved in Japan, which indirectly stimulates chloride secretion via activation of CaCCs [27–29]. Diquafosol submitted New Drug Approval (NDA) to the FDA in 2003 but was denied FDA approval following phase 3 clinical trial where it failed to meet its primary endpoints of reduced corneal staining and increased corneal clearing [30]. A phase 4 clinical trial for diquafosol is currently recruiting. An anti-absorptive drug in development, P-321 (SHP-659), is an ENaC inhibitor that increased tear production in normal and DED mice [31, 32]. A phase 2 clinical trial from 2016 failed to show a significant difference between P-321 and placebo on various outcome measures. Notably, ENaC inhibition with amiloride demonstrated minimal electrophysiological effect in our recent in vivo human pilot study, which may explain the apparent lack of efficacy of P-321 in the USA clinical trials [7••]. See Table 2 for additional details on therapeutic ocular surface ion transport targets.

## Experimental Approaches

A variety of mechanisms are used to measure ion transport and elucidate the location of ion transporters on the ocular surface. Likewise, a variety of clinical tests are used to measure the tear film.

## Ocular Surface Potential Difference (OSPD) Measurement

Clinical evaluation of ocular surface health typically involves slit lamp examination with fluorescein and lissamine green (LG) staining, tear breakup time (TBUT), Schirmer’s test, corneal sensation, MMP-9 levels, and tear fluid osmolarity. As a direct measure of ocular surface function, we exploited the millivolt (mV) electrical potentials generated by ion transport across the ocular surface epithelium. The concept of ocular surface potential difference (OSPD) was originally introduced for studies of sodium and chloride transport in experimental animals [16, 72], and more recently, applied to humans [7••]. The OSPD signal arises from the activities of various ion transporters in ocular surface epithelia, as depicted in Fig. 1A and Table 1. By manipulating experimental conditions, such as using selective transport modulators (activators or inhibitors), ion substitution, or genetic knockout/knockdown, it is possible to systematically characterize the ion transport pathways in vivo. Quantitative interpretation of OSPD data can be facilitated by mathematical modeling [16]. The OSPD method has broad potential applications in studying normal ocular surface physiology and disease, characterizing ocular surface ion transport pathways, and testing investigational drugs in animals and humans.

OSPD is measured using a high-impedance voltmeter/bioamplifier connected to a computer system using an analog-to-digital converter. A head stage is used to connect the measuring and reference electrodes to the bioamplifier. The measuring electrode contacts the ocular surface using a perfusion catheter whose tip is immersed in fluid bathing the ocular surface while the reference electrode is inserted subcutaneously. Solution exchange is accomplished using a gravity or peristaltic pump perfusion system (Fig. 1B). We adapted the system to measure OSPD in human subjects in which the head is stabilized using a slit lamp, with the tip of the perfusion catheter positioned under direct visualization in a fluid pocket created by eversion of the lower lid without contacting the ocular surface [7••]. Unlike nasal PD studies that often have considerable noise due to difficulty achieving and maintaining optimal measuring electrode positioning, the OSPD system provides a low-noise, robust signal that is related quantitatively to the activities of ion transport pathways at the ocular surface.

Our group has used OSPD measurements from sequential perfusion exchanges to study chloride transport pathways on the ocular surface (Fig. 1C). After determination of baseline OSPD using a high chloride solution that mimics the tear film, five solution exchanges are done to isolate ENaC, CFTR, and CaCCs functions. A high chloride solution containing the ENaC inhibitor, amiloride, produces minimal depolarization, suggesting minimal ENaC activity. A low chloride solution that probes basal transcellular and paracellular chloride transport pathways produces a rapid, modest hyperpolarization. Adding the cAMP agonist, forskolin, produces a more gradual but larger hyperpolarization due to activation of CFTR and potentially other cAMP-dependent ion channels. The potent and selective CFTR inhibitor, CFTRinh-172, produces a rapid and near complete reversal of the forskolin-induced hyperpolarization. Finally, the calcium agonist, ATP, produces a rapid hyperpolarization followed by slow depolarization due to transient elevation in cytoplasmic calcium, which activates CaCCs and calcium-activated potassium channels.

## Short-Circuit Current (Isc) Measurement

The transport of ions from the basolateral and apical sides of epithelial membranes creates a transepithelial voltage (V_te_), generally recorded using an Ussing chamber. Measurements of V_te_ are often referred to as open-circuit recordings and can be useful for studying the secretory and absorptive channels such as CFTR and ENaC, respectively. When V_te_ is held constant at 0 mV, it is possible to measure the charge flow, known as short-circuit current (Isc) [73]. In animal corneal and conjunctival specimens, Isc demonstrated the activating and inhibiting effects to forskolin and CFTRinh-172, respectively, on CFTR channels [27, 74, 75].

## Whole-Cell Patch-Clamp Measurement

Whole-cell patch clamp measurements yield biophysical information about single ion channel function by characterizing current flow through voltage and ligandgated ion channels. These measurements offer functional information regarding specific pharmacological agents by capturing the effect on ionic current following their administration [76]. Recordings are obtained by inserting a glass pipette into the cell membrane and applying suction, forming a seal between the pipet and membrane thus creating the whole-cell configuration. Pipettes contain an ionic solution that mimics the intracellular environment and connects to a recording electrode [77]. After achieving whole-cell configuration, cells are pulsed with hyperpolarizing and depolarizing voltages to induce currents. This technique was recently utilized to identify the modulatory activity of novel CFTR activator compounds relative to that produced by maximal forskolin activation [64•].

## cAMP Measurement

As a secondary messenger, cAMP has numerous downstream effects including CFTR-mediated chloride secretion and activation of protein kinase A (PKA). Therefore, cAMP measurements offer insight into the mechanism of therapeutic agents on the ocular surface. Effective measurements can be obtained using commercial kits [78]. These kits utilize a competitive enzyme immunoassay which can be used to measure cAMP levels in tear fluid, ocular surface epithelial cells, and the lacrimal gland [66].

## Immunohistochemistry (IHC)

Immunohistochemistry (IHC) utilizes antibodies to target and localize antigens in a particular tissue [79]. IHC can localize ion transporters to the apical or basolateral membranes of epithelial cells based on the pattern of staining. For example, using immunofluorescence microscopy, chloride channels such as CFTR and Clc-2 have been identified in the apical membrane of human corneal epithelium [18].

## Tear Film Osmolarity

The ocular environment needs regulated tear flow, which is driven by osmolarity. Measurements of tear film osmolarity, usually acquired by measuring the freezing point depression or tear fluid electrical conductivity, have been historically difficult to acquire [80]. Despite the challenge of measuring tear osmolarity, tear hyperosmolarity is a hallmark feature of DED [81]. Tear osmolarity is considered one of the best predictors of DED severity when compared to Schirmer’s test, meibomian gland grading, TBUT, and corneal and conjunctival staining [82, 83]. Measurements have been facilitated through development of technologies such as the I-PEN Tear Osmolarity System (I-MED Pharma: Quebec City, Canada). This device detects and measures tear film osmolarity on orbital tissues bathed in tear film, such as the palpebral conjunctiva, in approximately two seconds. Another method for determining tear film osmolarity is the TearLab Osmolarity System (TearLab: San Diego, CA), which requires only 50 nL of tear film to determine osmolarity. Both systems indirectly measure tear film osmolarity based on electrical impedance.

## Tear Volume

Schirmer’s test evaluates aqueous tear production. The test is carried out by applying paper strips to the inferior temporal aspect of both conjunctival sacs of the patient. After 5 minutes, the length of the wetted paper is measured [84]. A measurement of less than 5 mm of strip wetting after 5 minutes is diagnostic for aqueous deficiency [85••]. The phenol red thread (PRT) test is less invasive than Schirmer’s test. It involves placing a soft thread treated with phenol red (phenolsulfonphthalein), a pH indicator, on the ocular surface for 15 seconds. The thread will turn red when contacted by alkaline tears, and the length of the red (wet) portion of the thread is measured. A length of 20 mm in 15 seconds is considered normal and anything less than 11 mm is criteria for DED [84]. Another effective way to quantitatively gauge aqueous production is the measurement of the tear meniscus height and cross-sectional volume of tears. Tear meniscus heights between 0.1-0.2 mm indicate mild DED and values <0.1 mm indicate moderate to severe DED [85••].

## Corneal Fluorescein Staining

Corneal fluorescein staining provides insight into the severity of DED and the structural condition of the epithelium by highlighting loss of tight junctions. Fluorescein is a dark orange dye applied to the lower cul-de-sac of the eye and distributed across the ocular surface via blinking. The eye is subsequently examined under cobalt blue light where the density and extent of staining are assessed [64•]. Staining is commonly scored with the Oxford or National Eye Institute (NEI) scale [86].

## Animal Models

Animal models are useful to elucidate the pathology of DED and develop therapeutic agents. Dry eye can be experimentally induced in mice via subcutaneous injection of 5 mg/mL of scopolamine hydrobromide three times per day for 14 days while placed in a desiccating environment with continuous airflow (15 L/min), 35% humidity, and a constant temperature of 25°C [64•]. Using this scopolamine-induced DED model, tear volume levels are significantly reduced with resultant increased corneal staining scores [64•, 65•]. Another method of inducing DED is lacrimal duct cautery (LDC). The extraorbital lacrimal gland is exposed via linear skin incisions and the lacrimal duct is ablated with high temperature cautery. LDC produces a DED model with an instant and marked decrease in tear volume with concomitant increase in corneal staining [22•, 23]. Models can also be induced through genetic knockdown of AQP expressed in the corneal epithelium, specifically AQP1, AQP3, and AQP5 [87, 88].

In addition to DED mouse models described, there are also multiple mouse models specific to SS. Mice deficient in thrombospondin-1 (TSP-1), a matricellular glycoprotein that modulates cell migration and plays a critical role in wound healing, develop SS and the accompanying ocular surface dryness [89–91]. TSP-1 mice lines are generated through homologous recombination in embryonic stem cells that disrupt TSP-1 genes [92]. A recent review evaluated multiple SS animal models and determined the non-obese diabetic (NOD) model, which demonstrates decreased glandular secretion and lymphocytic infiltration, to be most optimal for studying SS pathogenesis and drug testing [89, 93].

## Conclusion

DED remains a major unmet need, with a significant USA and global prevalence. Current therapies primarily target inflammation despite tear film homeostasis and osmolarity playing key etiological roles. The modulation of ion transporters on the ocular surface epithelium therefore represents an attractive target for the development of pro-secretory and anti-absorptive therapies that aim to increase tear fluid secretion. We have summarized the current limited knowledge of ocular surface ion transport mechanisms and promising ion transport-related therapeutic targets. The full extent of ion transport mechanisms on the ocular surface remains to be elucidated. Methods such as OSPD measurements are exciting novel approaches to further investigate and identify ion transporters and their respective modulators.

## Figures and Tables

**Table 1:** Ocular surface ion transporters

	Gene	Transporter name	Location	Putative function
1	TRP	TRPV1, TRPV2, TRPV4, TRPA1, TRPM8	Apical	Non-selective cation channel
	ASIC	ASIC		Acid-sensing cation channel
2	SLC8A1	NCX	Apical	Na^+^/Ca^2+^ exchanger
3	SLC10A6	SOAT	Apical	Na^+^-dependent organic anion transporter
4	ATP2B1/ATP2C1	Mg^2+^/Ca^2+^-ATPase	Apical	ATP-activated transport of Ca^2+^ and Mg^2+^
5	CFTR	CFTR	Apical	cAMP-activated Cl^−^ channel
	CLCN2	Clc-2		
6	P2RY2	P2Y2	Apical	Purinergic GPCR, indirectly activates CaCC via increasing intracellular Ca^2+^
7	CLCA	CaCC	Apical	Ca^2+^-activated Cl^−^ channel
8	SCNN1A/G/B	ENaC	Apical	Amiloride-sensitive Na^+^ channel
9	KCN	K_v_	Apical	Voltage-gated K^+^ channel
10	ATP12A	H^+^/K^+^-ATPase	Apical	ATP-activated exchange of H^+^/K^+^
11	AQP5	AQP-5	Apical/Basolateral	Water channel protein*
12	SLC12A1	NKCC	Basolateral	Electroneutral Na^+^/K^+^/2Cl^−^ cotransporter
13	ATP1A1/ATP1B1	Na^+^/K^+^-ATPase	Basolateral	ATP-activated exchange of 3 Na^+^/2 K^+^
14	SLC9A1	NHE	Basolateral	Na^+^/H^+^ exchanger
15	SLC12A6	KCC	Basolateral	K^+^/Cl^−^ cotransporter
16	SLC4	CBE	Basolateral	Cl^−^/HCO_3_^−^ exchanger
17	KCNQ	K_v_7	Basolateral	cAMP-activated K^+^ channel
18	KCNN	KCa	Basolateral	Ca^2+^-activated K^+^ channel

* Not an ion transporter, but critical for movement of water

**Table 2:** Dry eye disease ion transport therapies

Therapy name	Mechanism of action	Clinical trial		Relevant references
		Identifier	Phase	
Diquafosol	Purinergic P2Y2 receptor agonist	NCT04668118	4	[8••, 33, 34•, 35••, 36–49]
Tivanisiran (SYL1001)	siRNA TRPV1 inhibitor	NCT04819269	3	[35••, 50, 51]
ALY688	Adiponectin receptor agonist	NCT04899518	3	[52–54]
RGN-259 (Tb4)	ATP-responsive purinergic receptor P2X4 agonist	NCT03937882	3	[35••, 55–57]
VSJ-110	CFTR activator	NCT04622345	2	[22•, 23–25]
P-321 (SHP-659)	ENaC inhibitor	NCT02824913, NCT02831387	2	[32, 58•]
AR-15512	TRPM8 agonist	NCT04498182	2	[59–63•]
Cact-3	CFTR activator	NA	NA	[64•]
Isorhamnetin	CFTR activator	NA	NA	[65•]
Cryosim-3 (C3)	TRPM8 agonist	NA	NA	[8••, 61]
Pituitary Adenylate Cyclase-Activating Peptide (PACAP)	Increases cAMP, PKA, and AQP-5	NA*	NA*	[8••, 66, 67•, 68–70]
2-Guanidine-4-Methylquinazoline (GMQ)	ASIC3 agonist	NA	NA	[8••, 71]

* No DED-related clinical trial
